# Molecules, magic and forgetful fruit flies: the supernatural science of medical gas research

**DOI:** 10.1186/2045-9912-1-23

**Published:** 2011-09-06

**Authors:** George Mychaskiw

**Affiliations:** 1 Department of Anesthesiology, Drexel University College of Medicine, 245 N. 15th Street, MSC-310, Philadelphia, Pennsylvania 19102, USA

## Abstract

Medical gas research often involves the study of molecules under extraphysiologic conditions, that is, conditions that do not exist in nature. This "supernatural" nature of medical gas research sometimes produces results that appear to be almost "magic" to those schooled in traditional physiology

*"Any sufficiently advanced technology is indistinguishable from magic"*.

-Arthur C. Clarke

## 

Often times in the day to day work of scientific endeavor, our efforts are not so much discovery as window dressing. Innovation has become a casualty of increasingly limited resources and the demands for concrete results in return for investment. Thus, the National Institutes of Health (NIH) tend to fund known investigators in established project lines and we learn more and more about vanishingly small and irrelevant details of things such as the thromboelastographic characterization of fibrinolysis, while the incidence of autism in the United States stands at 1 in 110 children and continues to increase without explanation [1]. Similarly, commercial scientific funding emphasizes the certain result, that is, the "win", rather than a gamble on the unknown and studies of Pharma drug × vs. drug Y predominate, but studies of drug × on an untested medical condition are far less common, that is, nonexistent. Even in the Food and Drug Administration (FDA), hallmark terms are "non-inferior" and "substantially equivalent", with the path for approval of the unique product in an unmet area of therapeutic need is extraordinarily arduous, to the point of killing new technologies before they are born.

In a sense, scientific innovation has been the victim of the expansion of our body of knowledge. In an earlier era, all investigation broke new ground and any research could be considered "high risk", as there was no assurance of a useful result. So, we have reached a point where we understand just enough about the known universe to limit the exploration of new territory. After all, if we are comfortable in our neighborhood, why go traveling across town to unfamiliar areas? Indeed, basic research has become so arcane that we have developed a new term, "translational", to emphasize the practical application of these arcane findings. We find this to be a curious sort of lexography; after all, if research is truly relevant, does it need translation? In our quest for the "sure thing" we struggle to find meaning for the meaningless. One need look only to the expansion of medical knowledge in the decade 1960-1970 compared to 2000-2010 as a demonstration of where we are (not) going. Could an innovation such as cardiopulmonary bypass be fostered in the contemporary era and is it any wonder that the delineation of the human genome has yielded little tangible result?

Hyperbaric oxygen (HBO) is one of these "frontier" therapies that, despite showing clinical promise, is widely discounted except for a very small and limited set of indications (e.g. decompression illness, carbon monoxide poisoning), without evidence justifying the negative view of the medical community, (including the Undersea and Hyperbaric Medical Society, which is loathe to consider applications of HBO outside of 13 "approved indications) [2]. Many otherwise reasonable clinicians and scientists automatically dismiss any usefulness of HBO for "off-label" conditions, such as stroke, concussion and autism, even when presented with well-conducted studies demonstrating effectiveness. Much of this has to do with the "supernatural" nature of oxygen under pressure and remarkable results that appear as "magic" to those versed in conventional science. The "supernatural" nature of HBO is not the stuff of ghosts and goblins, but rather, the behavior of a natural substance under extraphysiologic conditions. Normal human physiology takes place at or near normal atmospheric pressure, so the behavior of a familiar molecule, such as oxygen, under "supernatural" conditions is not only unpredictable, but also potentially efficacious in completely unexpected ways. Clinically, HBO appears to offer benefit in multiple neurologic conditions including autism, stroke and concussion [3,4]. Some results, such as the amelioration of neonatal asphyxia with one rescue treatment [5], are so unexpected that they appear to be magic. HBO is not magic; it is simply the behavior of a molecule in a mileu outside of human experience. Recall that the physiology of many organisms at extremes of temperature and pressure, such as deep-dwelling sea creatures, appeared initially to be strange and unusual "magic".

All of this would be fodder for quiet academic fireside discussion, were it not for the very real patients whose suffering could be helped by a treatment, HBO, that is ignored because of its foreign and unexpected results. After all, we were taught in medical school that neurologic damage is permanent, brain cells do not lay dormant and they certainly do not regenerate and yet, all of these axioms have been found to be untrue, but sometimes under unusual, "supernatural" conditions. Sports fans may be familiar with Dave Duerson. A formidable defensive player, Duerson was widely acclaimed for his ability to deliver devastating blows and sack quarterbacks. Unsuccessful following his professional career, he committed suicide earlier this year and asked that his brain be examined for damage sustained as a result of concussions sustained throughout his career. Duerson was indeed found to have chronic traumatic encephalopathy (CTE) that may have caused his depression and cognitive dysfunction. It is the clinical impression of physicians using HBO that the therapy is extraordinarily effective for CTE and has benefited many who have the means to find and fund the therapy (personal communication, Pete Stephens, MD). These physicians are greatly frustrated and become emotional at the thought of the suffering that could be eliminated and the natural history of CTE changed, were the therapy accepted and reimbursed by third party payers, such as Medicare. At our institution, we have started an observational study of the effectiveness of HBO in CTE, including a search for objective biomarkers, but skepticism of the "supernatural" effects of HBO dictates that it will be quite some time before this therapy becomes "standard of care". Similarly, HBO is showing clinical promise in the treatment of autism [4], but is widely considered to be just slightly above quackery. Again, we are taught that autism is a condition that has to be managed and cannot be treated. At Drexel we have also started down that long road to "standard of care" and evaluating the role of HBO in autism, using investigational biomarkers. Parents and caregivers, however, know and see the remarkable improvement in some autistic children following HBO treatment and do not care to wait for the scientific establishment to pronounce the therapy validated. Without the validation, unfortunately, the therapy is not reimbursed and the promise of a better way of life for their children is limited to those able to shoulder the expense.

Finally, our veneration of the established limits our ability to discard the "proven" technologies in use, even when they present a hazard to health and safety. The volatile anesthetic gases are an example. Sevoflurane is the most commonly used volatile anesthetic gas, especially in pediatric anesthesia, where it has nearly 100% of the market. It enjoys the advantages of pleasant smell, ease of use, cardiovascular stability and relatively low blood solubility. Previous to its introduction in 1992, pediatric anesthesia was dominated by halothane, which was troubled by a propensity for cardiac arrhythmias, increased incidence of hepatitis and high blood solubility leading to prolonged time to emerge from anesthesia. It is not surprising, therefore, that, once introduced, sevoflurane quickly replaced halothane. Unfortunately, that is not the end of the story. Recent animal studies have conclusively demonstrated that all the NMDA receptor antagonists, including sevoflurane, cause widespread neurodegeneration and brain damage in infants [6,7]. In our laboratories, we are evaluating the influence of anesthetic exposure in a developing drosophila, that is, fruit fly model. Initially, it appears that flies exposed to sevoflurane become "forgetful", mimicking the behavior of flies that have been genetically modified to express proteins found in human Alzheimer's disease (unpublished data). Normal fruit flies climb the walls of a glass jar when inverted; "forgetful" fruit flies do not. Additionally, there is some clinical evidence that sevoflurane is associated with a higher incidence of postoperative delirium, seizures and prolonged cognitive dysfunction in humans [8-10]. More worrisome, there are some studies in both animals and humans suggesting that exposure to anesthetics at an early age correlates with a diagnosis of neurodevelopmental diseases later in life, including learning disabilities and autism [7,11]. This is not a mystery to the FDA and has been the subject of two review panels. Alternative anesthetics that seem to be less neurotoxic, such as desflurane and propofol, are available and safe in children, but they are more technically difficult to administer. So, despite this accumulation of "soft" evidence, there is an unwillingness to abandon sevoflurane, leading the FDA to make the confusing recommendation that, while there is not enough evidence to recommend a change in the existing practice of sevoflurane use, anesthesia should be avoided in children under age 2 [12]. The veneration of the established permits the unrestricted use of sevoflurane, while the incidence of autism in the United States has continually increased without explanation since 1992, the year of sevoflurane's introduction to the market. Coincidence? Possbily. Statistics are funny things and, given a large enough database, the incidence of autism can probably be shown to correlate with the incidence of electric car use in California. So this may all be a statistical accident and the correlation meaningless. Absence of evidence, however, is not evidence of absence and it is also possible that sevoflurane is the thalidomide of the new millennium. Our laboratories are not only further examining the statistical correlations, we are also investigating the role of HBO in the prevention and treatment of anesthetic-induced neurodegeneration.

We therefore welcome with great excitement and enthusiasm the Journal of Medical Gas Research. As we have discussed, medical gases, such as HBO and sevoflurane, can manifest unusual and unexpected behavior at the molecular level. The atmospheric "phlogiston" of the pre-scientific era may still excite as we examine the unknown, the "magic" behavior of the natural under "supernatural" conditions. There is no more thrilling feeling than to experience a completely unexpected and unexplainable result of a scientific study. In medical gas research, we are all pioneers of a sort and we congratulate the readership of the new journal on joining in our adventure. Those who wish to closely examine the 15^th ^variation of the 22^nd ^step of fibrinolysis may be best served at another venue. Those who want to experience at least some portion of the feeling that Neil Armstrong had with that "one small step", need only read on.

Philadelphia, PA

June, 2011

## Competing interests

Dr. Mychaskiw is a member of the professional speaker bureaus of Baxter Healthcare, Inc. and GE Healthcare, Inc. He has also received research support from and is a consultant to Baxter Healthcare, Inc and HBT-USA, LLC. The opinions expressed in this article are those of the author and do not necessarily represent the opinions of the Undersea and Hyperbaric Medical Society, the Drexel University College of Medicine or Tenet Healthcare, Inc.
